# Impaired blood‐brain barrier in the microbiota‐gut‐brain axis: Potential role of bipolar susceptibility gene *TRANK1*

**DOI:** 10.1111/jcmm.16611

**Published:** 2021-05-20

**Authors:** Jianbo Lai, Jiajun Jiang, Peifen Zhang, Caixi Xi, Lingling Wu, Xingle Gao, Yaoyang Fu, Danhua Zhang, Yiqing Chen, Huimin Huang, Yiyi Zhu, Shaohua Hu

**Affiliations:** ^1^ Department of Psychiatry the First Affiliated Hospital Zhejiang University School of Medicine Hangzhou China; ^2^ The Key Laboratory of Mental Disorder Management in Zhejiang Province Hangzhou China; ^3^ Brain Research Institute of Zhejiang University Hangzhou China; ^4^ Zhejiang Engineering Center for Mathematical Mental Health Hangzhou China; ^5^ Wenzhou Medical University Wenzhou China

**Keywords:** bipolar disorder, blood‐brain barrier, gut microbiota, *TRANK1*, type 1 interferon

## Abstract

Bipolar disorder (BD) is a common psychiatric illness with high prevalence and disease burden. Accumulating susceptibility genes for BD have been identified in recent years. However, the exact functions of these genes remain largely unknown. Despite its high heritability, gene and environment interaction is commonly accepted as the major contributing factor to BD pathogenesis. Intestine microbiota is increasingly recognized as a critical environmental factor for human health and diseases via the microbiota‐gut‐brain axis. BD individuals showed altered diversity and compositions in the commensal microbiota. In addition to pro‐inflammatory factors, such as interleukin‐6 and tumour necrosis factor‐α, type 1 interferon signalling pathway is also modulated by specific intestinal bacterial strains. Disruption of the microbiota‐gut‐brain axis contributes to peripheral and central nervous system inflammation, which accounts for the BD aetiology. Administration of type 1 interferon can induce the expression of *TRANK1*, which is associated with elevated circulating biomarkers of the impaired blood‐brain barrier in BD patients. In this review, we focus on the influence of intestine microbiota on the expression of bipolar gene *TRANK1* and propose that intestine microbiota‐dependent type 1 interferon signalling is sufficient to induce the over‐expression of *TRANK1*, consequently causing the compromise of BBB integrity and facilitating the entrance of inflammatory mediators into the brain. Activated neuroinflammation eventually contributes to the occurrence and development of BD. This review provides a new perspective on how gut microbiota participate in the pathogenesis of BD. Future studies are needed to validate these assumptions and develop new treatment targets for BD.

## 1 INTRODUCTION

Bipolar disorder (BD) is a serious, recurring and highly disabling affective mental disorder.^1^ According to data released by the World Health Organization in 2011, the global lifetime prevalence of BD is 2.4%.^1^ The suicide risk of BD patients is much higher than that of the general population, and the proportion of suicide deaths among BD individuals is as high as 6% to 7%.^2^ BD frequently occurs in the adolescence and early adulthood, severely impairing the cognitive and social functions of patients, and causes a huge burden of disease to the family and society.^3^ However, the pathogenesis of BD remains largely unclear. The existing diagnostic criteria for BD are mainly based on clinical symptoms and lack of objective biomarkers. In clinical practice, it can easily cause missed diagnosis and misdiagnosis, which is not conducive to the treatment and prognosis of patients.^3^ Therefore, further exploring the pathogenesis and exploring the targeted markers of the disease is of great significance for the clinical diagnosis and treatment of BD.

The interaction of genetic and environmental factors is considered to be one of the major aetiology of BD.^4^ Different environmental factors (such as early childhood trauma, stress events, pathogen infection and intestinal ecosystem imbalance) act on genetically susceptible individuals, hindering the normal growth of neurons and promoting neuroinflammation, metabolism disturbance and oxidative stress, leading to dysfunction of the brain areas related to emotion regulation, and eventually causes emotional and behavioural abnormalities.^4^ However, it is still unclear how environmental factors and genetic factors influence each other and contributes to the onset of BD. In the current review, special attention is paid to the bipolar risk gene, etratricopeptide repeat and ankyrin repeat containing 1 (*TRANK1*) and its potential interaction with the gut microbiota, which is speculated to impair the integrity of blood‐brain barrier (BBB) and facilitates the neuroinflammation in BD.

## 2 *TRANK1*, A RISK GENE FOR BD

Epidemiological studies of twins have shown that the genetic heritability of BD is as high as 70%‐85%, suggesting that genetic factors play an important role in the pathogenesis of BD.^5^, ^6^ In previous studies, we also found that specific single nucleotide polymorphisms were associated with the age of onset for BD patients.^7^ In recent years, several important large‐sample whole‐genome sequencing studies have suggested that *ADCY2, ANK3, CACNA1C, TENM4, SYNE1, ERBB2, TRANK1*, etc, may be the risk genes for BD.^8^, ^9^, ^10^ In a newly published study, *TRANK1* has been further confirmed as the susceptibility gene for BD.^11^ The *TRANK1* gene is located on the short arm of chromosome 3 (3p22.2). The autoantibody of TRANK1 protein was originally found in the brain tissue of a mouse model of systemic lupus erythematosus. It is mainly produced and secreted by immune cells such as neutrophils and natural killer cells in the peripheral blood and is widely expressed in the cerebral cortex, including the hippocampus, amygdala and cerebellum.^12^ It is worth noting that the expression level of *TRANK1* gene in the postmortem brain tissue of BD patients was significantly higher than that of healthy controls.^13^ Compared with healthy individuals, circulating anti‐TRANK1 IgG was increased in a group of 356 plasma samples from patients with schizophrenia.^14^ An integrated analysis of mRNA co‐expression network revealed that genes highly correlated with *TRANK1* were significantly involved in the biological processes related to synaptic plasticity, dendritic spine, axon guidance and circadian rhythm.^11^ In addition, *TRANK1* rs71947856 variant was associated with circadian regulation and reported birth difficulties in patients with Kleine‐Levin syndrome, which is a rare disease characterized by severe episodic hypersomnia, with cognitive impairment and apathy or disinhibition.^15^ However, the specific function of the *TRANK1* gene in BD has not been clarified so far.

Previous studies have shown that *TRANK1* may be involved in immune‐related signal regulation. In a mouse model where the interferon (IFN) regulatory factor 7 was knocked out or up‐regulated, it was found that the change in the expression level of this regulatory factor can affect the transcription level of *TRANK1* through the type 1 IFN signalling pathway.^16^ In differentiated hepatocytes, IFN‐α can up‐regulate the expression of *TRANK1* through the STAT/JAK signalling pathway.^17^ In a socially isolated mouse model, it was found that the expression level of the *TRANK1* gene in the prefrontal lobe was significantly increased, and the expression of matrix metalloproteinase 9 (MMP9) and plasma membrane vesicle‐associated protein‐1 (PV‐1) was up‐regulated, which indicated that the integrity of the blood‐brain barrier (BBB) was impaired, and the permeability was increased.^18^ Elevation in pro‐inflammatory factors, such as interleukin‐6 (IL‐6) and tumour necrosis factor‐α (TNF‐α) in the brain, was also observed and further confirmed the activation of the central immune reaction.^18^ Based on these findings, it is plausible to speculate that the expression of *TRANK1* is regulated by the IFN signalling pathway, and the *TRANK1* gene may be an important mediator affecting BBB permeability and neuroinflammation in the brain. However, how the expression of *TRANK1* gene is regulated under pathological processes of BD remains to be further studied.

## 3 DISTURBANCE OF INTESTINAL MICROBIOTA IN BD

Recent researches have taken an increasing focus on the microbiota‐gut‐brain (MGB) axis regulation in neuropsychiatric diseases.^19^ On the one hand, microorganisms inhabiting in the intestine, such as bacteria, fungi, viruses and bacteriophages, can synthesize and release various metabolites, including small molecule hormones, inflammatory mediators, neurotransmitters and short‐chain fatty acids, which send ascending signals to the brain through the endocrine, immune and vagus nerve pathways.^19^ Notably, the vagal transmission from the gut to the brain influences monoaminergic systems and is crucial for various bodily functions, such as mood regulation and immune response.^20^, ^21^ The vagus nerve plays as an immune modulator of intestinal homeostasis via three different approaches, including the hypothalamus‐pituitary‐adrenal (HPA) axis, the splenic sympathetic anti‐inflammatory and the cholinergic anti‐inflammatory pathways.^20^ Therefore, although the vagal transmission may not directly influence the expression of *TRANK1*, its regulation on the intestinal immune responses may link gut microbes to host gene expression. On the other hand, stress in particular, along with other factors, such as diet choice and the HPA regulation, represents up‐to‐bottom regulation of the gut microbiota.^19^, ^22^ The bidirectional communication of the MGB axis is now considered to play an important role in maintaining physical and mental well‐being.^22^, ^23^

Preliminary studies have shown that the diversity and structure of the intestinal microbiota of BD patients are different from that of healthy individuals.^24^, ^25^ A previous study has reported that the faecal abundance of *Faecalibacterium* and *Ruminococcaceae* decreased in the BD patients, and the abundance of *Faecalibacterium* was negatively correlated with the severity of depression.^24^ The diversity of gut microbiota in BD patients is negatively correlated with the course of the disease. The abundance of *Actinobacteria* and *Coriobacteria* in the intestine was increased, and the abundance of *Lactobacillus* was positively correlated with serum IL‐6, lipid, tryptophan levels and other indicators.^25^ In recent years, our research team has also shown great interest in gut microbiota and has carried out a serious of studies to investigate its characteristics in depressed patients. Using 16sRNA sequencing, we reported gut microbial changes in diversity and compositions in BD depression.^26^ Compared with healthy controls, untreated depressed BD patients showed decreased diversity in gut microbiota and reduced abundance of butyrate‐producing bacteria,^26^ which may foster systemic inflammation in BD patients.^27^ Based on specific bacterial operational taxonomic units, microbial markers could be established to distinguish depressed BD patients from healthy controls and predict one‐month treatment outcome of quetiapine monotherapy.^26^ Moreover, we reported gut microbiol signatures can effectively distinguish unipolar and bipolar patients during depressive episodes.^28^ In addition, we have systematically reviewed the research advances on gut microbiota in mood disorders and have proposed future research directions in this field.^27^, ^29^, ^30^ Given the limited number of studies, small to moderate sample sizes, different ethnicity and regions, uncontrolled clinical characteristics and medications across studies, findings in these studies have both similarities and differences, and thus should be interpreted carefully.

Above research results suggest that the disturbance of intestinal microbiota may be an important environmental factor in the onset of BD. However, it is still unclear how the gut microbiota affects the MGB axis, which in turn leads to abnormal mood and behaviour in BD individuals.

## 4 IMPAIRED BLOOD‐BRAIN BARRIER FUNCTION IN BD

The BBB is an important barrier for the exchange of substances and nutrients between the peripheral circulation and the central nervous system (CNS) and plays a key role in maintaining normal physiological functions of the brain.^31^ Previous studies have suggested that oxidative stress and chronic inflammation also exist in patients with BD, which may lead to the destruction of the integrity of the BBB, but the specific pathological mechanism remains unclear.^32^ A recent study has shown that in BD patients with manic episodes or remission, the levels of claudin‐5 and zonulin in peripheral blood were increased.^33^ Using qRT‐PCR and immunohistochemistry, another study reported that the expression of claudin‐5, a tight junction protein, was reduced in the hippocampus of individuals diagnosed with major psychiatric disorders and was correlated with disease duration and age at onset.^34^ In addition, blood levels of soluble intercellular adhesion molecule‐1 (ICAM‐1), a transmembrane glycoprotein, were elevated in BD patients in a state‐independent pattern.^35^ Higher soluble ICAM‐1 seems to be associated with altered permeability of the BBB. Thus, these preliminary studies indicated that the integrity of BBB in BD patients was damaged. Impaired BBB function in BD patients was also verified by contrast‐enhanced dynamic MRI scanning for quantitative assessment of BBB leakage, which was associated with more severe symptoms of depression and anxiety and longer course of illness.^36^

In recent years, studies have reported the influence of metabolites of intestinal microbiota on the BBB function, which may act as a protective or detrimental factor under different conditions.^37^, ^38^ In germ‐free mice, the expression of the tight junction proteins, occludin and claudin‐5, was lower compared with those with a normal glora. Germ‐free adult mice exposed to gut microbiota from pathogen‐free mice can reverse the increased BBB permeability and up‐regulate the expression of tight junction proteins.^39^ In addition, propionate produced by bacterial metabolism can reduce the damage caused by oxidative stress to BBB through a CD14 cell‐dependent mechanism.^40^ While in patients with multiple sclerosis, the interaction between the disturbed intestinal microbiota and the BBB can even affect the course of disease.^37^ In adult rhesus monkeys, antibiotic treatment caused changes in gut microbial compositions, especially a decrease in acetic acid‐ and propionic acid‐producing phyla and genera, accompanied by the increase in BBB permeability.^41^ Intestinal microorganisms can produce a variety of microbial‐associated molecular patterns (MAMPs), such as bacterial lipopolysaccharide (LPS), lipoprotein and double‐stranded RNA. On the one hand, as a result of the "leaky gut", LPS produced by the intestinal microbiota can enter the blood circulation through the intestinal mucosal barrier, and interact with the Toll‐like receptor 4 (TLR4) on the surface of immune cells, such as peripheral macrophages and neutrophils, activating the downstream signalling pathway of TLR4 and releasing inflammatory factors including IL‐1β, IL‐6 and TNF‐α. These inflammatory factors can enter the CNS through the compromised BBB. On the other hand, as a result of the impaired BBB function in BD patients, LPS in the blood circulation can also directly penetrate the BBB and bind to the TLR4 on the surface of microglia, which then activates the neuroinflammation process in the CNS.^42^ A recent published study showed that the levels of neuroinflammation and neurodegeneration in the brain of TLR4 knockout mice were significantly alleviated, suggesting that the inhibition of the LPS‐TLR4 signalling pathway could be an alternative strategy to alleviate gut microbiota‐associated neuroinflammation.^43^ Therefore, changes in diversity and compositions of gut microbiota, and even its metabolome, may serve as underlying source for both systemic inflammation and neuroinflammation.

Notably, mounting evidence has confirmed the close relationship between BD and immune dysfunction.^44^ BD immunology is an evolving field concerning non‐infectious inflammatory alterations in the peripheral and CNS.^44^ In a previous study, we reported elevated serum IL‐6 levels in depressed BD patients, accompanied with abnormalities in T‐cell sub‐populations.^45^ Other pro‐inflammatory cytokines including TNF‐α, soluble TNF‐type 1 receptor, IL‐2 receptor, IL‐1β and C‐reactive protein were also elevated in patients with acute depressive or manic episodes.^44^, ^46^ When the pro‐inflammatory messages enter into the CNS, brain microglia is activated and induces neuroinflammtion. Under inflammatory conditions, the metabolic process of kynurenine turns towards the neurotoxic quinolinic acid pathway rather than the neuroprotective kynurenic acid pathway,^30^ thus disrupting the neural growth and neurotransmitter production, and contributing to the abnormalities in mood and behaviours.

Taken together, the above findings indicate that intestinal dysbiosis may be related to the impairment of BBB integrity. BBB dysfunction facilitates the entrance of peripheral inflammatory mediators into the CNS, thus promoting neuroimmune response and participating in the occurrence and development of BD.

## 5 INTESTINAL MICROBIOTA REGULATE *TRANK1* EXPRESSION VIA TYPE 1 IFN SIGNALLING

The interaction between microorganisms and host genome has been designated as one of the major research directions of the Human Microbiology Project.^47^ Intestinal microbes are involved in the regulation of host development and physiological processes, including the formation of various organs, energy metabolism and host immune response.^48^ However, disturbances of the intestinal microbiota may also participate in the occurrence and development of diseases by affecting the expression of host genes.^49^, ^50^ For example, the complex interaction between the host genome and the intestinal microbiota may affect the age of onset and clinical manifestations of patients with inflammatory bowel disease.^49^ In BD patients, it was also found that the diversity of intestinal commensal bacteria was negatively correlated with the methylation level of cg05733463 on the biological clock gene *ARNTL*.^50^ However, few studies have ever explored the relationships between gut microbiota and susceptibility genes of BD.

**FIGURE 1:**
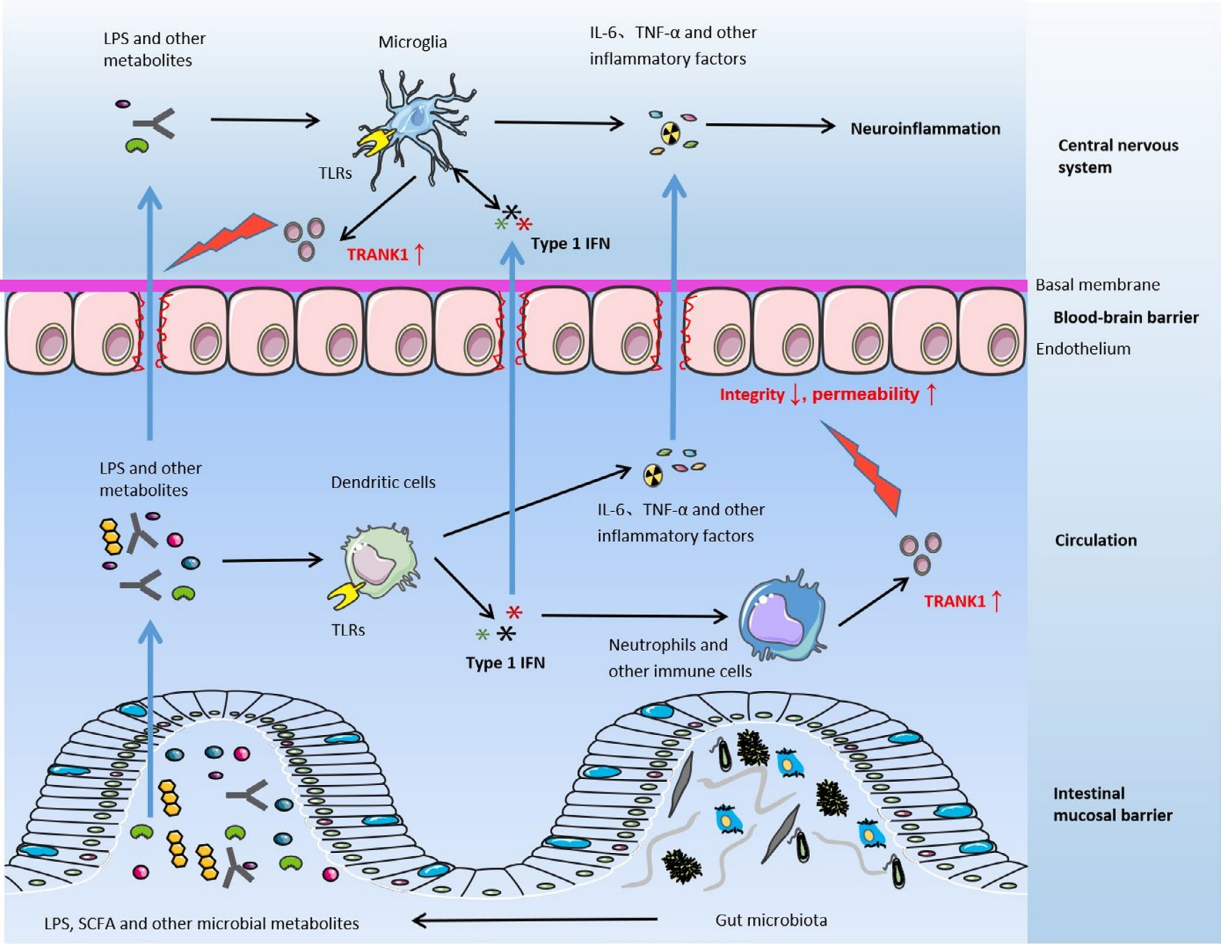
Gut microbiota‐dependent type 1 IFN signalling induces *TRANK1* expression and BBB impairment. Compared with healthy individuals, the diversity and compositions of the intestinal microbiota of BD patients is altered. Various metabolites produced by the commensal microbiota, such as bacterial lipopolysaccharide (LPS), short‐chain fatty acids (SFCA) and other metabolites, can enter the peripheral circulation through the intestinal mucosal barrier. Circulating bacterial antigens, such as LPS, are recognized by the Toll‐like receptors on the surface of dendritic cells and stimulate the release of cytokines including type 1 IFN, IL‐6, TNF‐α and other inflammatory mediators. Type 1 IFN stimulates neutrophils and other immune cells to synthesize and secrete TRANK1 protein. TRANK1 protein in the peripheral circulation acts on the BBB, resulting in decreased integrity and increased permeability of the BBB. Various inflammatory mediators are more easily to enter the central nervous system and activate microglia to induce neuroinflammation, which is involved in the pathogenesis of BD

In addition to the regulatory role of gut microbiota on pro‐inflammatory factors, such as IL‐1β, IL‐6 and TNF‐α, type 1 IFN pathway is also modulated by the signals from the commensal micriobiota.^51^ As a pleiotropic family of cytokines, type 1 IFN plays as a critical mediator of host immune response to intestine‐derived antigens, including bacteria and virus.^51^ However, only some specific bacterial strains have been confirmed to induce type 1 IFN production in vitro studies.^52^, ^53^ Double‐stranded RNA from the intestinal lactic acid bacteria triggered IFN‐β production from murine bone marrow‐derived dendritic cells (DCs) via a TLR3‐dependent pathway.^52^ Two specific *L acidophilus* strains, rather than other probiotic strains, were able to induce IFN‐β production from murine bone‐marrow‐derived DCs in a TLR2‐dependent manner,^53^ which could be inhibited by *Bifidobacterium bifidum*.^54^ In mice deficient in autophagy proteins, responses to intestinal bacterial pathogen *Citrobacter rodentium* were enhanced by type 1 IFN‐dependent signalling.^55^ Interestingly, a fungus, *Debaryomyces hansenii*, was found to impair mucosal healing in Crohn's disease intestinal tissue through the myeloid‐specific type 1 IFN‐CCL5 pathway.^56^ Moreover, type 1 IFN signalling was involved in prostaglandin E2‐induced intestinal inflammation via disrupting the microbiota‐regulatory T‐cell communication.^57^ Therefore, the type 1 IFN signalling pathway manifests as an important coordinator of human immune response to the intestinal microbiota. As shown in previous studies, type 1 IFN signalling is sufficient to induce the expression of *TRANK1* gene.^16^, ^17^ Overexpression of *TRANK1* was associated with impaired BBB function, as revealed by increased levels of MMP9 and PV‐1.^18^ These findings together indicate that gut microbiota‐dependent type 1 IFN signalling may up‐regulate the expression of *TRANK1* and further damaged the integrity of the BBB, thus increasing its permeability and exacerbating the neuroinflammatory basis of BD (Figure 1).

## 6 CONCLUDING REMARKS

Despite advances in biotechnology, the underlying pathogenesis of BD remains a mystery. A series of susceptibility genes of BD have been identified, but the exact functions of these genes have been rarely claimed. In this brief review, we provide a new perspective to investigate the role of these genes by paying special attention to *TRANK1* and its interaction with human intestine microbiota, which is also recognized as a critical environmental factor for human health.^58^ The immune pathway is an important approach that the gut and the brain communicate bidirectionally. Indeed, the peripheral inflammation and neuroinflammation in the CNS is a distinguished hallmark of BD and may have a close relationship with the gut microbial compositional alterations. Increasing evidence has revealed that gut microbiota can regulate local and systemic inflammation via the type 1 IFN signalling. Herein, we propose that the gut mircobiota‐derived type 1 IFN signalling is essential in regulating the host expression of *TRANK1* gene, which participates in the destruction of BBB integrity and facilitates CNS inflammatory processes. Over‐activated neuroinflammation thereby promotes the occurrence and development of BD.

More evidence is needed to confirm this speculation mechanistically. For example, germ‐free mice receiving faecal microbiota transplantation from critically ill BD patients should be constructed to verify the disease‐like phenotypes, inflammatory status, expression level of *TRANK1* and markers of impaired BBB. Whether inhibition of the type 1 IFN signalling can down‐regulate the expression of *TRANK1* and protect the BBB integrity remains to be studied. In human tissues, although up‐regulated expression of *TRANK1* in postmortem brain from BD patients has been observed,^13^ the expression level of *TRANK1* in the peripheral circulation also needs to be explored. Researches in *TRANK1* knockout animals can help to elucidate the gene function and its interaction with the gut microbiota.

Overall, it is intriguing that if the expression of bipolar susceptibility genes can be regulated by targeting the gut microbiota, microorganism‐derived metabolites or downstream type 1 IFN signalling pathway. Better understanding the role of gut microbiota in human health and diseases helps to elucidate the pathogenesis of BD and provides novel treatment strategies.

## CONFLICT OF INTEREST

The authors confirm that there are no conflicts of interest.

## AUTHOR CONTRIBUTIONS

**Jianbo Lai:** Conceptualization (lead); Writing‐original draft (lead). **Jiajun Jiang:** Conceptualization (equal); Writing‐original draft (equal). **Peifen Zhang:** Conceptualization (equal); Writing‐original draft (equal). **Caixi Xi:** Conceptualization (equal); Writing‐original draft (equal). **Lingling Wu:** Conceptualization (equal); Writing‐original draft (equal). **Xingle Gao:** Conceptualization (equal); Writing‐original draft (equal). **Yaoyang Fu:** Conceptualization (equal); Writing‐original draft (equal). **Danhua Zhang:** Conceptualization (equal); Writing‐original draft (equal). **Yiqing Chen:** Conceptualization (equal); Writing‐original draft (equal). **Huimin Huang:** Conceptualization (equal); Writing‐original draft (equal). **Yiyi Zhu:** Conceptualization (equal); Writing‐original draft (equal). **Shaohua Hu:** Conceptualization (lead); Supervision (lead); Writing‐review & editing (lead).

## Data Availability

Data sharing not applicable – no new data generated.
